# Protein tyrosine kinase 2: a novel therapeutic target to overcome acquired EGFR-TKI resistance in non-small cell lung cancer

**DOI:** 10.1186/s12931-019-1244-2

**Published:** 2019-12-02

**Authors:** Xuexia Tong, Ryosuke Tanino, Rong Sun, Yukari Tsubata, Tamio Okimoto, Mayumi Takechi, Takeshi Isobe

**Affiliations:** 1 Department of Internal Medicine, Division of Medical Oncology & Respiratory Medicine, Faculty of Medicine, Shimane University, 89-1 Enya-cho, Izumo, Shimane 693-8501 Japan; 2 Department of Respiratory and Critical Care Medicine, General Hospital of Ningxia Medical University, Yinchuan, Ningxia China; 3 Department of Experimental Animals, Interdisciplinary Center for Science Research, Organization for Research and Academic Information, Shimane University, Izumo, Shimane Japan

**Keywords:** Combined inhibition, Drug resistance, EGFR, PTK2, Tyrosine kinase inhibitor

## Abstract

**Background:**

Protein tyrosine kinase 2 (PTK2) expression has been reported in various types of human epithelial cancers including lung cancer; however, the role of PTK2 in epidermal growth factor receptor (EGFR)-mutant non-small cell lung cancer (NSCLC) has not been elucidated. We previously reported that pemetrexed-resistant NSCLC cell line PC-9/PEM also acquired EGFR-TKI resistance with constitutive Akt activation, but we could not find a therapeutic target.

**Methods:**

Cell viability in EGFR-mutant NSCLC cell lines was measured by the WST-8 assay. Phosphorylation antibody array assay for receptor tyrosine kinases was performed in PC-9 and PC-9/PEM cell lines. We evaluated the efficacy of EGFR and PTK2 co-inhibition in EGFR-TKI-resistant NSCLC in vitro. Oral defactinib and osimertinib were administered in mice bearing subcutaneous xenografts to evaluate the efficacy of the treatment combination in vivo. Both the PTK2 phosphorylation and the treatment combination efficacy were evaluated in erlotinib-resistant EGFR-mutant NSCLC cell lines.

**Results:**

PTK2 was hyperphosphorylated in PC-9/PEM. Defactinib (PTK2 inhibitor) and PD173074 (FGFR inhibitor) inhibited PTK2 phosphorylation. Combination of PTK2 inhibitor and EGFR-TKI inhibited Akt and induced apoptosis in PC-9/PEM. The combination treatment showed improved in vivo therapeutic efficacy compared to the single-agent treatments. Furthermore, erlotinib-resistant NSCLC cell lines showed PTK2 hyperphosphorylation. PTK2 inhibition in the PTK2 hyperphosphorylated erlotinib-resistant cell lines also recovered EGFR-TKI sensitivity.

**Conclusion:**

PTK2 hyperphosphorylation occurs in various EGFR-TKI-resistant NSCLCs. Combination of PTK2 inhibitor and EGFR-TKI (defactinib and osimertinib) recovered EGFR-TKI sensitivity in the EGFR-TKI-resistant NSCLC. Our study result suggests that this combination therapy may be a viable option to overcome EGFR-TKI resistance in NSCLC.

## Introduction

Lung cancer is the leading cause of cancer-related mortality worldwide with non-small cell lung cancer (NSCLC) being the largest subgroup. It accounts for approximately 85% of all lung cancers [1]. Epidermal growth factor receptor (EGFR) tyrosine kinase inhibitors (TKIs) have been used to treat *EGFR* mutation-positive NSCLC. The response rate reported was ≤80% and progression-free survival (PFS) was ~ 10–14 months [2, 3]. However, most tumors initially responding to EGFR-TKIs eventually recur as they acquire resistance [4, 5]. Platinum-based chemotherapy is used as second-line therapy, whereas pemetrexed or docetaxel is used as third-line therapy in NSCLC patients in the event of disease progression after first-line EGFR-TKI therapy. They showed a median PFS of 6.4 months and a median overall survival of 19.2 months as salvage chemotherapies [6, 7]. Overcoming EGFR-TKI resistance is important for prolonging overall survival. Though considerable effort has been made, ~ 18–30% of resistance mechanisms have not yet been elucidated [8–10].

Various acquired resistance mechanisms to EGFR-TKIs have been reported over the past decade. The most common factor of alternative signaling is the hepatocyte growth factor-MET pathway. It explains 5–10% of all acquired resistance [8, 11, 12]. Other bypass pathways include the amplification of ErbB family genes [13, 14], IGF1R [15], and AXL [16]. PIK3CA mutation, the loss of PTEN, epithelial-to-mesenchymal transition, and small-cell transformation are also associated with acquired resistance to EGFR-TKI in NSCLC [17]. Osimertinib is a third-generation EGFR-TKI; it targets EGFR T790 M mutation-positive tumors. It has demonstrated superior efficacy compared to first and second generation EGFR-TKIs. Moreover, it was more efficacious than standard first-line EGFR-TKIs in advanced EGFR-mutated NSCLC but had a similar safety profile and lower incidences of serious adverse events [18]. Although osimertinib is clinically efficacious, acquired resistance to it is inevitable. Mechanisms of acquired resistance to osimertinib in patients with EGFR T790 M mutations include the C797S mutation in *EGFR* exon 20, the reduction or disappearance of T790 M, activation of alternative pathways, and phenotypic alterations [19, 20]. However, few studies have reported on osimertinib-resistant cases. Furthermore, most mechanisms of acquired osimertinib resistance remain unclear and are largely responsible for treatment failure [20, 21]. Thus, the emergence of acquired resistance to EGFR-TKIs is alarming and requires investigation. Understanding the molecular mechanism of EGFR-TKI resistance may aid in the development of potential treatment options for tumors with acquired EGFR-TKI resistance.

Protein tyrosine kinase 2 (PTK2) or focal adhesion kinase is a member of the non-receptor protein tyrosine kinase family [22, 23]. It regulates cell survival, proliferation, migration, invasion, and adhesion via scaffolding and kinase activity [24]. *PTK2* expression has been explored in several human epithelial cancers including breast, ovarian, colorectal, and lung cancers. *PTK2* upregulation is associated with malignancy, metastasis, and poor survival [25]. PTK2 may be a prognostic marker and a novel molecular target for cancer treatment options. PTK2 inhibitors effectively inhibited cancer growth in vitro and in vivo [26, 27]. Several clinical studies have been initiated on PTK2 inhibitors for patients with solid tumors [28–30].

The aim of this study was to elucidate the mechanisms of acquired resistance to EGFR-TKIs in NSCLC cell lines. We previously found that an EGFR-activated, pemetrexed-resistant NSCLC cell line acquired EGFR-TKI resistance and had constitutive Akt signaling activation compared with the parental cell line [31]. In this study, we explored the molecular mechanisms of EGFR-TKI resistance in the pathways upstream from Akt. Herein, we show the potential of PTK2 as a therapeutic target for acquired resistance to EGFR-TKIs and provide evidence that the combination of a PTK2 inhibitor and an EGFR-TKI is a potentially efficacious therapy for EGFR-TKI-resistant NSCLC.

## Material and methods

### Cell culture and reagents

The human lung adenocarcinoma cell line PC-9 with an EGFR exon 19 deletion mutation (delE746_A750) was tested and authenticated by genetic testing in July 2016 using a PowerPlex® 16 STR system (Promega, Madison, WI). The pemetrexed-resistant cell line PC-9/PEM was established from its parental cell line PC-9 as described previously [31]. The PC-9/PEM clone1 monoclonal cell line was established from PC-9/PEM by seeding one cell per well of a 96-well plate. To establish erlotinib-resistant cell lines, PC-9 cells were exposed to gradually increasing concentrations of erlotinib. The dose was 3 nM at time zero and was incrementally raised to 30 μM over 6 months. The cell lines were named PC-9/ER-1 to PC-9/ER-6. The PC-9/OSI cell line was established by exposing PC-9 cells to stepwise increases in osimertinib concentration from 3 nM to 3 μM. The normal human lung tissue-derived cell line OUS-11 was purchased from JCRB Cell Bank (Osaka, Japan). The cells were cultured in RPMI-1640 growth medium (FUJIFILM Wako Pure Chemical Industries, Osaka, Japan) supplemented with 10% FBS and 50 μg/mL gentamicin at 37 °C in a humidified 5% CO2 incubator. Pemetrexed (Eli Lilly Japan, Hyogo, Japan) was diluted with PBS. Gefitinib, erlotinib, afatinib, osimertinib, PD173074, BLU-554, nintedanib, and defactinib were all purchased from Selleck Chemicals (Houston, TX) and diluted with DMSO (FUJIFILM Wako Pure Chemical Industries).

### Cell viability assay

Cell viability was determined by a 4-[3-(2-methoxy-4-nitrophenyl)-2-(4-nitrophenyl)-2H-5-tetrazolio]-l,3-benzene disulfonate sodium salt (WST-8) assay using Cell Counting Kit-8 (CCK-8; Dojindo Laboratories, Kumamoto, Japan). The IC_50_ was calculated using Prism v. 7.00 (GraphPad Software, San Diego, CA). Cells were seeded in a 96-well plate at a density of 2000–3000 per well and cultured with the indicated doses of drug-containing medium. Absorbances were measured on a Sunrise R microplate reader (Tecan Group, Männedorf, Switzerland) at 450 nm (reference wavelength was 630 nm). The absorbance for the blank well was subtracted from each absorbance value. The absorbance of each well was expressed as a percentage of growth relative to the untreated cells to determine the relative cell viability percentage.

### Reverse-transcription quantitative polymerase chain reaction (RT-qPCR)

Total RNA was extracted from cultured cells in a 6-well plate using the RNeasy Mini Kit (Qiagen, Hilden, Germany). The RNA was reverse-transcribed to cDNA using a ReverTra Ace qPCR RT Master Mix with gDNA Remover (Toyobo, Osaka, Japan) according to the manufacturer’s instructions. The primers, cDNA, and KOD SYBR qPCR Mix (Toyobo) were mixed and qPCR was performed in a Thermal Cycler Dice Real Time System II (TaKaRa Bio, Kusatsu, Shiga, Japan). The sequences for primers used are given in Additional file 1: Table S1. *GAPDH* was the normalization standard for relative expression. The qPCR was performed with a pre-denaturation step of 98 °C for 2 min and 40 cycles of 98 °C for 10 s and 68 °C for 30 s.

### MET copy number determination

Total DNA was extracted from the cells with a DNeasy Blood & Tissue Kit (Qiagen, Hilden, Germany). QuantiTect Multiplex PCR NoROX Master Mix (Qiagen), 100 ng DNA, TaqMan Copy Number Assays of *MET* (Hs01277655_cn) or *MAD1L1* (Hs00981515_cn) and *RPPH1* (Probe; 5′ CGTCCTGTCACTCCACTCCCATGTC 3′, forward primer; 5′ CGGAGGGAAGCTCATCAGTG 3′, reverse primer; 5′ CCCTAGTCTCAGACCTTCCCAA 3′) were mixed. *RPPH1* was the normalization standard for relative expression. The qPCR was performed with a denaturing step of 95 °C for 15 min and 45 cycles of 94 °C for 60 s and 60 °C for 60 s.

### Immunoblotting

Cells were lysed with M-PER (Mammalian Protein Extraction Reagent; Thermo Fisher Scientific, Waltham, MA, USA), with 1% (v/v) Phosphatase Inhibitor Cocktail (Nacalai Tesque, Kyoto, Japan), and 1% (v/v) Protease Inhibitor Cocktail (Thermo Fisher Scientific). Protein concentrations were determined with a Coomassie Plus Bradford Assay Kit (Thermo Fisher Scientific). Protein samples, Bolt LDS Sample Buffer (Thermo Fisher Scientific), and Bolt Sample Reducing Agent (Thermo Fisher Scientific) were mixed and heated at 95 °C for 5 min to denature the proteins. Total proteins were loaded onto a Bolt 4–12% Bis-Tris Plus Gel (Thermo Fisher Scientific). A Mini Gel Tank (Thermo Fisher Scientific) and a Mini Blot Module (Thermo Fisher Scientific) were used for electrophoresis. Proteins were transferred onto a ClearTrans Nitrocellulose Membrane (FUJIFILM Wako Pure Chemical Industries). Membranes were blocked with 5% non-fat milk for 1 h at room temperature (20–25 °C) and was incubated overnight at 4 °C with primary antibodies using Blocker BSA in TBS (Thermo Fisher Scientific). The following were primary antibodies: anti-EGFR, anti-pEGFR (Y1068), anti-PARP, anti-cPARP (D214), anti-pAkt (S473), anti-ERK1/2, anti-pERK1/2 (T202/Y204), anti-PTK2, anti-pPTK2 (Y566/567), anti-FGFR1 (D8E4), anti-pFGFR (Y653/654), anti-FGFR4 (D3B12) (Cell Signaling Technology, Danvers, MA, USA), anti-Akt (Santa Cruz Biotechnology, Dallas, TX, USA), and anti-β-actin (BioLegend, San Diego, CA, USA). Anti-Rabbit IgG, HRP-linked Whole Ab Donkey secondary antibody (GE Healthcare, Buckinghamshire, UK) was added at 1:3000 dilution for 1 h at room temperature. Immunoreactive bands were visualized using ECL Select Western Blotting Detection Reagent (GE Healthcare). Chemiluminescent signals on the membranes were acquired with LAS-4000 (Fujifilm, Tokyo, Japan). Density was calculated as the ratio of each intensity band quantified by ImageJ v. 1.8.0_112 (NIH, Bethesda, MD, USA).

### Phosphorylation antibody array for receptor tyrosine kinases

Human RTK Phosphorylation Antibody Array-Membrane (ab193662; Abcam, Cambridge, MA) was used to detect the phosphorylation of 71 receptor tyrosine kinases in PC-9 and PC-9/PEM cells according to the manufacturer’s instructions. Chemiluminescence signals from the membranes were acquired with LAS-4000 (Fujifilm).

### SiRNA transfection

Small interfering RNA (siRNA) assays were conducted using Silencer Select Negative Control siRNA (4,390,844; Thermo Fisher Scientific). Pre-Designed Silencer Select siRNA si#1 (s11485; Thermo Fisher Scientific) and si#2 (s11484; Thermo Fisher Scientific) were used against *PTK2*. Lipofectamine™ RNAiMAX reagent (1% (v/v); Thermo Fisher Scientific) and the siRNAs were dispersed in Opti-MEM medium (Thermo Fisher Scientific) in a 6-well plate at a final siRNA concentration of 15 nM and incubated for 30 min. PC-9/PEM clone1 cells were seeded at a density of 10^5^ per well. Cells were harvested 48 h after transfection and used in the subsequent experiments.

### Xenograft mouse model

All animal experimental protocols were approved by the Committee for Animal Experimentation of Shimane University, Shimane, Japan (No. IZ29–63). Female BALB/cA nu/nu mice aged 5 weeks were purchased from CLEA Japan (Tokyo, Japan). PC-9 and PC-9/PEM clone1 cells (2 × 10^6^) were injected subcutaneously into the left and right hind flanks, respectively, of 7-week-old mice. Two weeks later, the mice were randomly assigned to one of four groups (six mice per group) receiving vehicle, 25 mg/ kg/d defactinib, 5 mg/kg/d osimertinib, or 25 mg/kg/d defactinib plus 5 mg/kg/d osimertinib. Drugs were administered by oral gavage twice daily for 5 days per week. Tumor length and width were measured every 2–3 days using a caliper under the assumption that the tumors were hemi-ellipsoid using the formula: (π/9 × L^2^ × W), according to previous studies [32, 33].

### Sequence alignment

Blastp (protein-protein BLAST) was used to align Q05397 for the Query Sequence and P11362 for the Subject Sequence at the following URL: https://blast.ncbi.nlm.nih.gov/Blast.cgi?PROGRAM=blastp&PAGE_TYPE=BlastSearch&LINK_LOC=blasthome.

### Statistical analysis

Significant differences between pairs of treatment means were evaluated by a Student’s unpaired two-tailed *t*-test. Differences among > 2 groups were evaluated by one-way ANOVA and a post-hoc test. *P* < 0.05 was considered statistically significant. All data were analyzed in IBM SPSS Statistics v. 23 (IBM Corp., Armonk, NY).

## Results

### Validation of acquired EGFR-TKI resistance in PC-9/PEM cell line

**Fig. 1:**
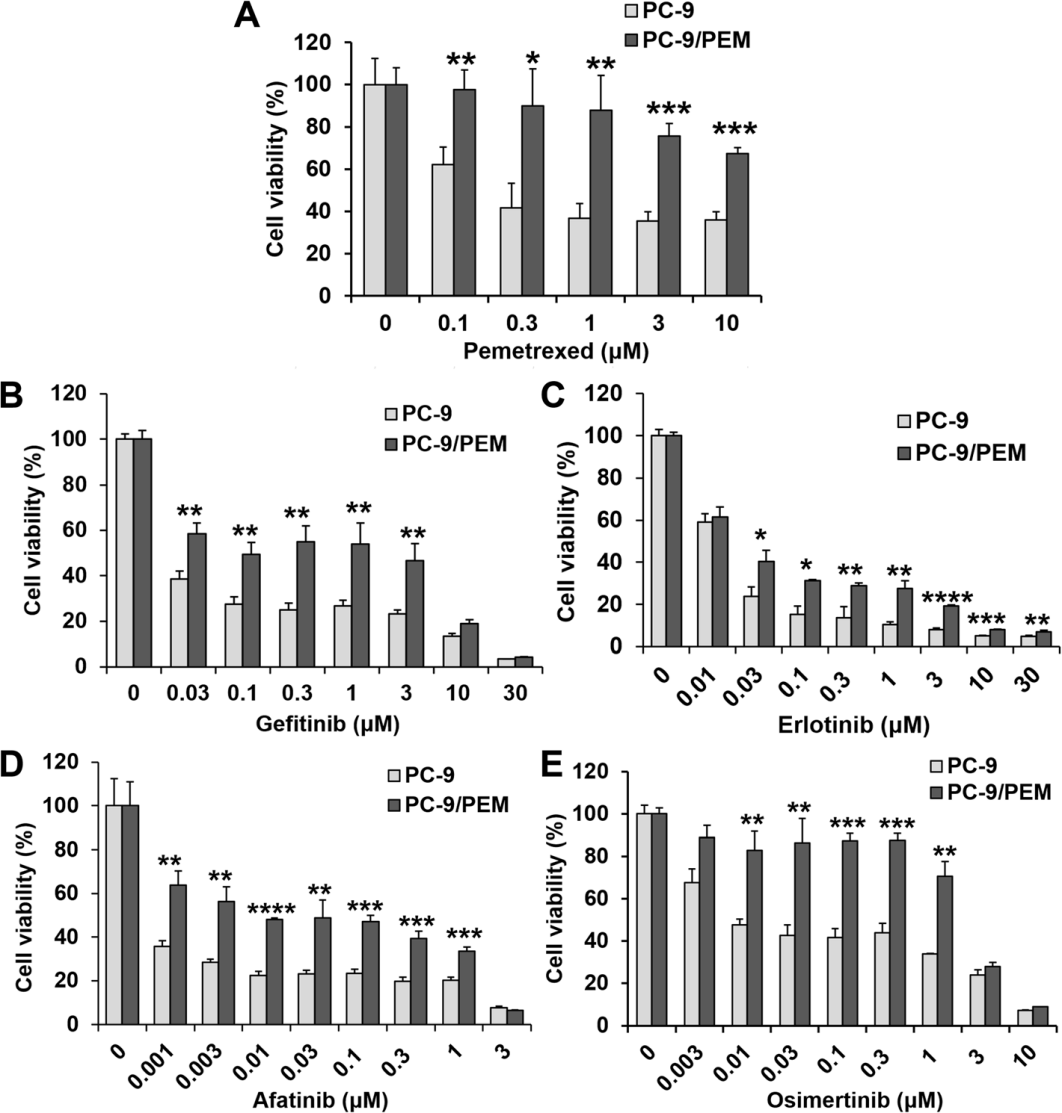
The pemetrexed-resistant PC-9 cell line acquired EGFR-TKI resistance. **a**-**e** Viabilities of the human EGFR-mutant NSCLC cell line PC-9 and the PEM-resistant cell line PC-9/PEM treated for 72 h with pemetrexed or EGFR-TKI at the indicated concentrations. Data are means (SD), *n* = 3. *, *P* < 0.05; **, *P* < 0.01; ***, *P* < 0.001; ****, *P* < 0.0001 by Student’s *t*-test

We used the WST-8 assay to assess drug resistance in PC-9/PEM for several EGFR-TKIs by comparing the viabilities of PC-9/PEM and PC-9 cells in response to exposures to pemetrexed or first- to third-generation EGFR-TKIs including gefitinib (first), erlotinib (first), afatinib (second), and osimertinib (third) (Fig. 1a-e). PC-9/PEM cells were relatively more resistant to pemetrexed (Fig. 1a) and all other EGFR-TKIs (Fig. 1b-e) compared to PC-9 cells.

### PC-9/PEM have hyperphosphorylated PTK2, which activates Akt signaling

**Fig. 2:**
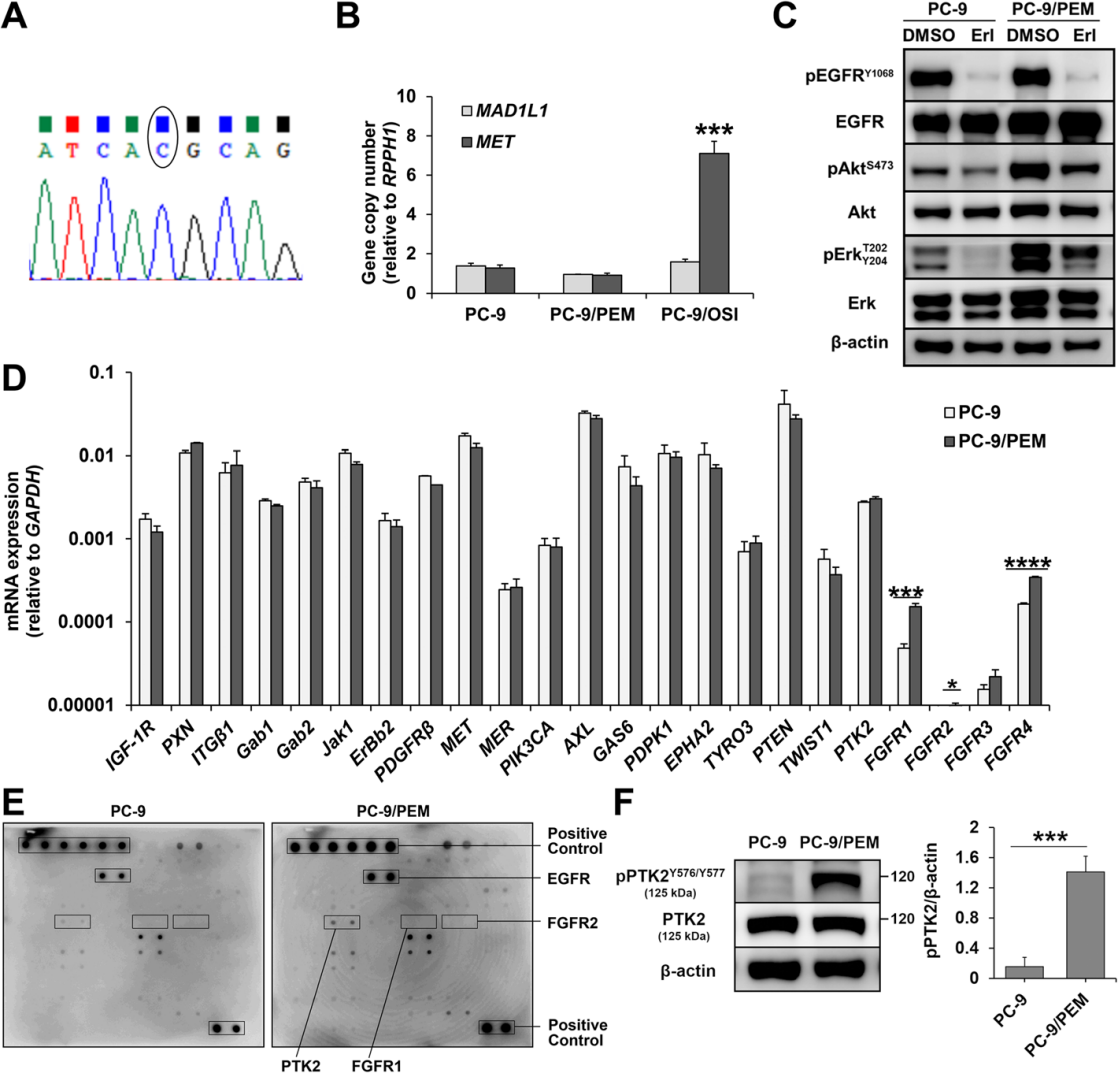
PC-9/PEM exhibits PTK2 hyperphosphorylation. **a** Direct sequencing chromatogram at the T790 site of EGFR in PC-9/PEM. **b** Copy number quantification of genomic DNA extracted from PC-9, PC-9/PEM, and PC-9/OSI. *MET* or *MAD1L1* copy numbers relative to *RPPH1* copy number are shown. *MAD1L1* was the negative control. Data are means (SD), *n* = 3. ***, *P* < 0.001 by Student’s *t*-test. **c** Immunoblots of the phosphorylated and total levels of the indicated proteins in PC-9 and PC-9/PEM cells treated with 1 μM erlotinib (Erl) or DMSO for 96 h. **d** Expressions of the indicated genes in PC-9 and PC-9/PEM cells. Data are means (SD), *n* = 3. *, *P* < 0.05, ***, *P* < 0.001, ****, *P* < 0.0001 by Student’s *t*-test. **e** Phosphorylation array analysis of 71 phosphorylated human receptor tyrosine kinases extracted from PC-9 and PC-9/PEM cells. **f** Representative immunoblots of phosphorylated and total PTK2 in PC-9 and PC-9/PEM cells and the ratio of phosphorylated PTK2 to β-actin. Data are means (SD), *n* = 3. ***, *P* < 0.001 by Student’s *t*-test

To confirm whether PC-9/PEM is genetically altered for EGFR-TKI resistance, we conducted direct sequencing of *EGFR*. No mutation was detected in the *EGFR* exons including T790 M of PC-9/PEM (Fig. 2a). We also evaluated the *MET* copy number as changes in *MET* confer acquired EGFR-TKI resistance in NSCLC. However, no *MET* amplification was detected in PC-9/PEM (Fig. 2b).

To elucidate the underlying EGFR-TKI resistance mechanisms in PC-9/PEM, we assessed Akt and Erk signaling downstream of EGFR in both PC-9 and PC-9/PEM cells after treatment with erlotinib or DMSO. As shown in Fig. 2c, erlotinib dramatically inhibited EGFR^Y1068^ phosphorylation in PC-9/PEM. Nevertheless, Erk^T202/Y204^ phosphorylation and especially Akt^S473^ phosphorylation were less inhibited in PC-9/PEM than they were in PC-9. To clarify the Akt hyperphosphorylation mechanism, we used RT-qPCR to measure gene expressions in the upstream signaling of the Akt pathway. Several fibroblast growth factor receptor (FGFR) family genes including *FGFR1* and *FGFR4* were significantly (*P* < 0.001) upregulated in PC-9/PEM compared to PC-9 (Fig. 2d). To assess the FGFR1 and FGFR4 protein expression and activation levels, we conducted immunoblotting analysis. Using anti-pFGFR^Y653/654^ antibody, we found a high-density band of phosphorylated protein in PC-9/PEM (Additional file 1: Figure S1A). Nonetheless, no FGFR1 or FGFR4 total protein bands were detected in either PC-9 or PC-9/PEM (Additional file 1: Figure S1A-C).

These results suggested that PC-9/PEM did not express FGFR1 or FGFR4. Anti-pFGFR^Y653/654^ antibody reacted with a phosphorylated protein (Additional file 1: Figure S1A). To identify the latter, we used phosphorylation array analysis to measure the phosphorylation levels of 71 receptor tyrosine kinases, including FGFR1 and FGFR2, in PC-9 and PC-9/PEM. The focal adhesion-associated protein kinase PTK2 was hyperphosphorylated in PC-9/PEM compared with PC-9 but FGFR1 and FGFR2 were not detected in both PC-9 and PC-9/PEM (Fig. 2e). We also use immunoblotting with anti-pPTK2^Y576/Y577^ antibody to confirm PTK2 hyperphosphorylation in PC-9/PEM relative to PC-9 (Fig. 2f). Therefore, PTK2^Y576/Y577^ hyperphosphorylation may account for the persistent Akt activation under EGFR inhibition in PC-9/PEM.

### The PTK2 inhibitor defactinib increased EGFR-TKI sensitivity by inhibiting Akt in PC-9/PEM

**Fig. 3:**
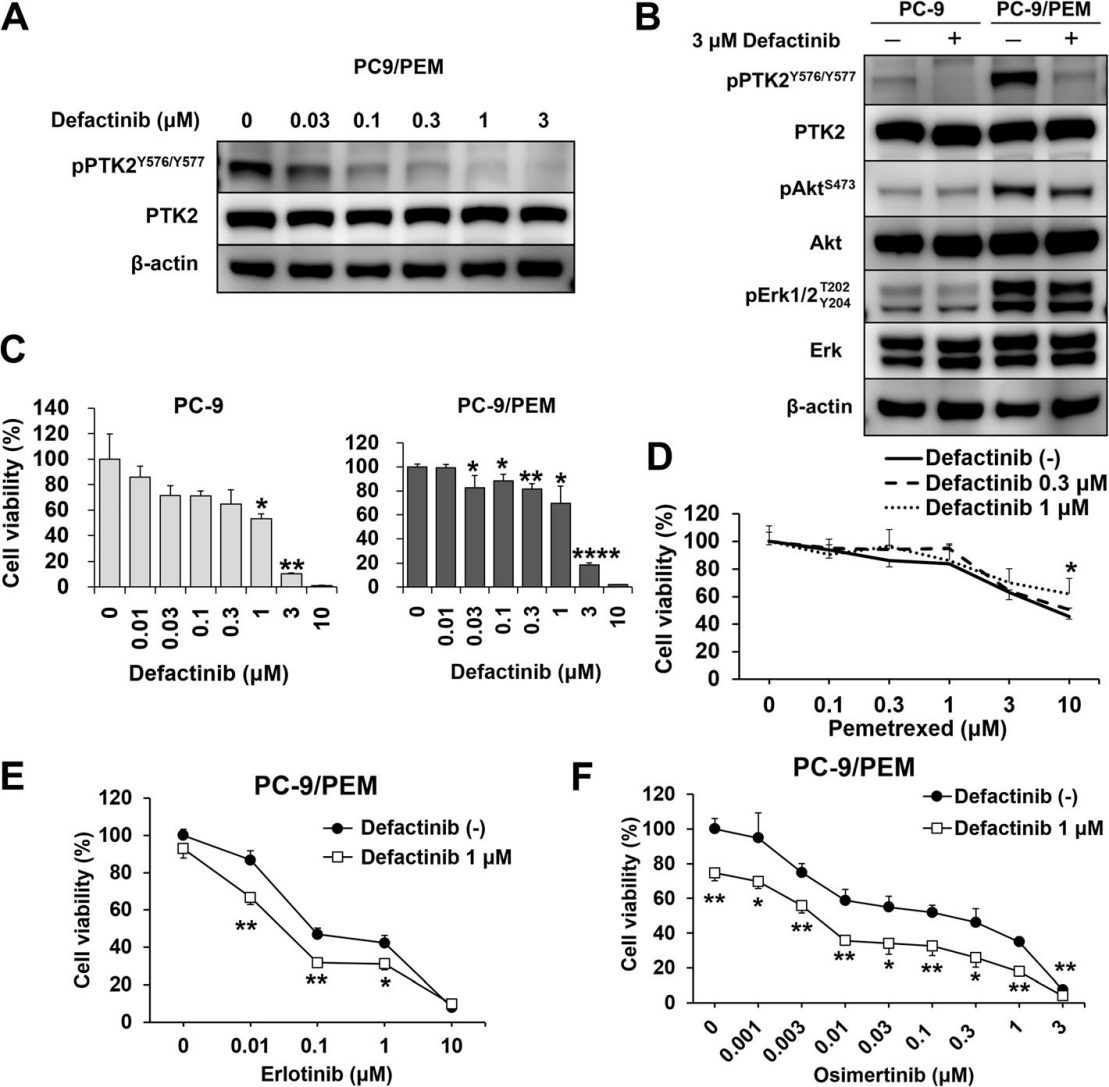
The PTK2 inhibitor defactinib recovers EGFR-TKI sensitivity, but not pemetrexed sensitivity, in PC-9/PEM. **a** Immunoblots of phosphorylated and total PTK2 in PC-9 and PC-9/PEM cells treated with defactinib at the indicated concentrations for 96 h. **b** Immunoblots of the phosphorylated and total levels of the indicated proteins in PC-9 and PC-9/PEM cells treated with 3 μM defactinib or DMSO for 96 h. **c** Viabilities of PC-9 and PC-9/PEM cells treated with defactinib at the indicated concentrations for 96 h. Data are means (SD), *n* = 3. *, *P* < 0.05; **, *P* < 0.01; ****, *P* < 0.0001. Data for treated and untreated cells were compared by one-way ANOVA and Tukey’s HSD multiple comparisons test. **d**-**f** Viabilities of PC-9/PEM cells either with or without defactinib and treated with pemetrexed (**d**), erlotinib (**e**), or osimertinib (**f**) at the indicated concentrations for 96 h. Data are means (SD), *n* = 3. *, *P* < 0.05; **, *P* < 0.01. **d** Data were compared by one-way ANOVA and Games–Howell multiple comparisons test. **e** and **f** Data were compared by Student’s *t*-test

To investigate the role of PTK2 in EGFR-TKI resistance in PC-9/PEM, cells were treated with defactinib. Immunoblotting showed that defactinib inhibited phosphorylation at the 576/577 tyrosine residues of PTK2 in a dose-dependent manner (Fig. 3a). Defactinib markedly suppressed PTK2^Y576/Y577^ phosphorylation in both PC-9 and PC-9/PEM. However, Akt^S473^ phosphorylation was decreased in PC-9/PEM but not in PC-9 (Fig. 3b). PC-9 and PC-9/PEM did not proliferate in the presence of 3 μM defactinib (Fig. 3c). The IC_50_ of defactinib for PC-9 and PC-9/PEM cells were 1.5 μM and 1.7 μM, respectively. Thus, PC-9/PEM and PC-9 were equally dependent on PTK2 for proliferation. For PC-9/PEM cells, combination therapy with pemetrexed and defactinib did not have any synergistic effect (Fig. 3d). In contrast, co-treatment with defactinib and erlotinib (Fig. 3e) or osimertinib (Fig. 3f) had restored sensitivity to erlotinib and osimertinib on PC-9/PEM compared with either treatment alone; moreover, the combination (PTK2 inhibitor and EGFR-TKI) was relatively more efficacious against EGFR-TKI-resistant cells with PTK2 hyperphosphorylation.

### FGFR1 inhibitor PD173074 inhibits PTK2 phosphorylation

It was previously thought that PC-9/PEM cells had FGFR^Y653/654^ phosphorylation (Additional file 1: Figure S1A); we treated the cells with FGFR1 inhibitor PD173074 for validation that FGFR1 protein has no influence on the EGFR-TKI resistance. Surprisingly, immunoblotting with anti-pFGFR^Y653/654^ showed that PD173074 inhibited protein band phosphorylation in a dose-dependent manner but had no impact on EGFR phosphorylation (Additional file 1: Figure S2A). Immunoblotting with anti-pPTK2^Y576/577^ revealed that PD173074 also reduced the phospho-PTK2 protein bands (Additional file 1: Figure S2B). Similar with defactinib, PD173074 did not restore pemetrexed sensitivity (Additional file 1: Figure S2C). However, the combination of erlotinib and PD173074 had a synergistic effect on PC-9/PEM cells (Additional file 1: Figure S2D). Erlotinib had no effect on PTK2 phosphorylation while PD173074 alone slightly induced Akt phosphorylation, however, a combination of both repressed Akt phosphorylation (Additional file 1: Figure S2E). The combination also upregulated the apoptotic marker cleaved PARP in PC-9/PEM (Additional file 1: Figure S2F). Thus, the EGFR/Akt pathway plays a salvage role in response to PTK2/Akt pathway inhibition.

As the *FGFR4* gene expression was higher than that of *FGFR1* in both PC-9 and PC-9/PEM (Fig. 2d), we assessed the effects of the FGFR4 inhibitor BLU-554. BLU-554 alone did not inhibit PC-9 or PC-9/PEM proliferation (Additional file 1: Figure S2G and S2H). Furthermore, the combination of BLU-554 and EGFR-TKIs had no additive effect (Additional file 1: Figure S2I and S2J). We also tested the efficacy of the multiple tyrosine kinase inhibitor nintedanib (Additional file 1: Figure S2K). However, this agent provided no additive effect either (Additional file 1: Figure S2L). Regardless of the direct target protein of PD173074, the reaction of phosphorylated PTK2 indicated that PD173074 inhibits PTK2 of PC-9/PEM. Moreover, combination treatment of PD173074 and erlotinib induced apoptotic PC-9/PEM cells.

### Combination inhibition of PTK2 and EGFR enhances apoptosis in PTK2-activated monoclonal pemetrexed-resistant cell line

**Fig. 4:**
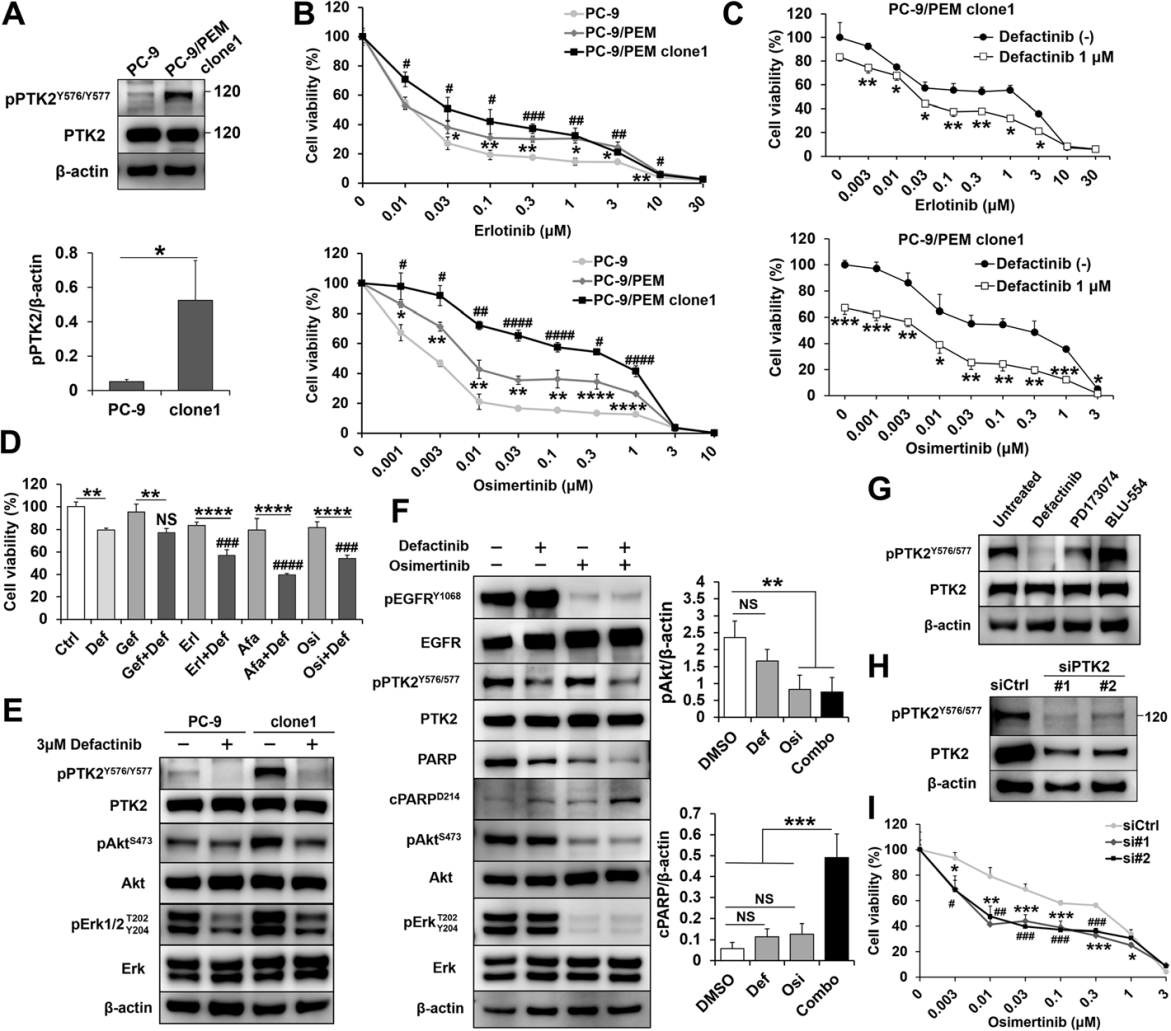
EGFR and PTK2 co-inhibition induce apoptosis in PC-9/PEM clone1. **a** Representative immunoblots of phosphorylated and total PTK2 in PC-9 and PC-9/PEM clone1 cells and the ratio of phosphorylated PTK2 to β-actin. Data are means (SD), *n* = 3. *, *P* < 0.05 by Student’s *t*-test. **b** Viabilities of PC-9, PC-9/PEM, and PC-9/PEM clone1 cells treated with erlotinib or osimertinib at the indicated concentrations for 96 h. Data are means (SD), *n* = 3. PC-9 vs. PC-9/PEM, *, *P* < 0.05; **, *P* < 0.01; ***, *P* < 0.001; ****, *P* < 0.0001, PC-9 vs. PC-9/PEM clone1, ^#^, *P* < 0.05; ^##^, *P* < 0.01; ^###^, *P* < 0.001; ^####^, *P* < 0.0001 by one-way ANOVA and Tukey’s HSD multiple comparisons test. **c** Viability of PC-9/PEM clone1 cells with or without defactinib and erlotinib (upper) or osimertinib (lower) at the indicated concentrations for 96 h. Data are means (SD), *n* = 3. *, *P* < 0.05; **, *P* < 0.01; ***, *P* < 0.001. Data for cells with and without defactinib were compared by Student’s *t*-test. **d** Viability of PC-9/PEM clone1 cells with or without 1 μM defactinib and EGFR-TKI (0.01 μM gefitinib, 0.03 μM erlotinib, 0.001 μM afatinib, or 0.01 μM osimertinib). Data are means (SD), *n* = 3. **, *P* < 0.01; ****, *P* < 0.0001, EGFR-TKI + Def vs. Def; ^###^, *P* < 0.001; ^####^, *P* < 0.0001, NS, not significant by one-way ANOVA and Tukey’s HSD multiple comparisons test. **e** Immunoblots of the phosphorylated and total levels of the indicated proteins in PC-9 and PC-9/PEM clone1 cells treated with 3 μM defactinib or DMSO for 96 h. **f** Representative immunoblots of the indicated proteins in PC-9 and PC-9/PEM clone1 cells with or without 1 μM defactinib (Def) and 0.1 μM osimertinib (Osi). Ratios of phospho-Akt to β-actin (upper) and cleaved PARP to β-actin (lower). Data are means (SD), *n* = 3. **, *P* < 0.01, ***, *P* < 0.001, NS, not significant by one-way ANOVA and Tukey’s HSD multiple comparisons test. **g** Immunoblots of phosphorylated and total PTK2 in PC-9/PEM clone1 cells treated with or without 1 μM defactinib, 1 μM PD173074 or 1 μM BLU-554 for 72 h. **h** Immunoblots of phosphorylated and total PTK2 in PC-9/PEM clone1 cells transfected with siRNAs against *PTK2*. The siCtrl was used as a negative control. **i** Viability of PC-9/PEM clone1 cells transfected with the indicated siRNAs and treated with osimertinib at the indicated concentrations for 96 h. Data are means (SD), *n* = 3. (siCtrl vs. si#1, *, *P* < 0.05; **, *P* < 0.01), (siCtrl vs. si#2, ^#^, *P* < 0.05; ^##^, *P* < 0.01) by one-way ANOVA and Tukey’s HSD multiple comparisons test

We examined the role of PTK2 activation to assess its dependency in EGFR-TKI resistance. We established several monoclonal cell lines derived from PC-9/PEM and selected PC-9/PEM clone1 as it had high PTK2 phosphorylation (Fig. 4a). PC-9/PEM clone1 had higher cell viability after treatment with erlotinib and osimertinib than PC-9/PEM or PC-9 (Fig. 4b). Defactinib recovered EGFR-TKI sensitivity in PC-9/PEM clone1 (Fig. 4c). We investigated whether the combination effect varies with the type of EGFR-TKI. To measure cell viability in the combination treatments, we set the concentration of each EGFR-TKI to ~IC_80_. The additive effect of defactinib differed among EGFR-TKIs (Fig. 4d). Defactinib most strongly sensitized afatinib and enhanced the efficacies of erlotinib and osimertinib but had no apparent influence on gefitinib potency.

To elucidate the profiles of the molecules downstream of the PTK2 axis, we evaluated the effects of PTK2 inhibition on downstream signaling. Complete PTK2 inhibition reduced the Akt phosphorylation level in PC-9/PEM clone1 to that of parental PC-9 (Fig. 4e). In contrast, there was insignificant difference in Akt phosphorylation in PC-9 under PTK2 inhibition by defactinib. Defactinib alone did not affect the phosphorylation of EGFR (Fig. 4f). Osimertinib downregulated pAkt^S473^ and pErk^T202/Y204^ more than defactinib via EGFR inhibition but it has no effect on inhibit pPTK2 in PC-9/PEM clone1. Although, osimertinib alone did not induce apoptosis, the combination treatment of defactinib and osimertinib significantly induced apoptosis (Fig. 4f), same as the combination of PD173074 and erlotinib (Additional file 1: Figure S2F). The FGFR inhibitor’s efficacy was confirmed in PC-9/PEM clone1. Our results show that FGFR1 inhibitor PD173074 downregulated PTK2 phosphorylation (Additional file 1: Figure S2B), while FGFR4 inhibitor BLU-554 did not (Fig. 4g). This is consistent with the cell viability assays using PC-9/PEM (Additional file 1: Figure S2D, S2J). These results suggested that PTK2 inhibition is an important factor in increasing the sensitivity to EGFR-TKI. In fact, siRNA knockdown of *PTK2* (Fig. 4h) sensitized PC-9/PEM clone1 to osimertinib (Fig. 4i). Moreover, the knockdown of *PTK2* resulted in decrease the band detected by anti-pFGFR^Y653/654^ antibody (Additional file 1: Figure S2M). This result indicated that the band detected by anti-pFGFR^Y653/654^ antibody was same as the band of PTK2 or PTK2 was the upstream protein of FGFR. The aforementioned findings indicated that PTK2 inhibition is a therapeutic target against EGFR-TKI-resistant NSCLC.

### Therapeutic efficacy of PTK2 inhibitor on xenograft tumor growth

**Fig. 5:**
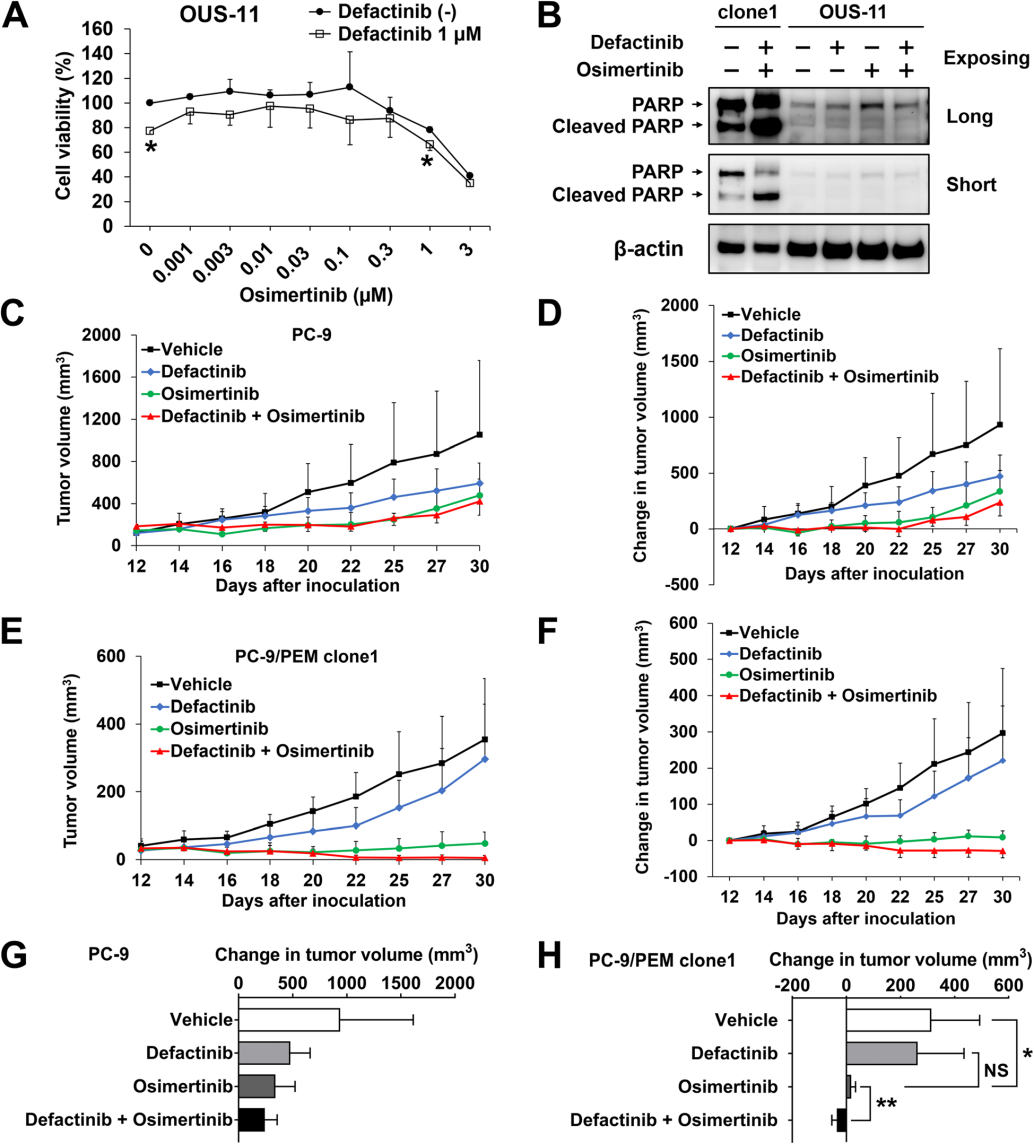
EGFR-TKI and PTK2 co-inhibition improved therapeutic efficacy in a xenograft model. **a** Viability of OUS-11 cells treated with 1 μM defactinib and osimertinib at the indicated concentrations for 96 h. Data are means (SD), *n* = 3. *, *P* < 0.05; ***, *P* < 0.001, by Student’s *t*-test. **b** Immunoblots of PARP and cleaved PARP in PC-9/PEM clone1 and OUS-11 cells with or without 1 μM defactinib (Def) and 0.1 μM osimertinib (Osi) following long and short exposing time. **c** and **e** Tumor volume of xenografts of PC-9 (**c**) and PC-9/PEM clone1 (**e**) were measured over time of the treatment after inoculation and the results are shown. Data are means (SD), *n* = 6. **d** and **f** Changes in tumor volumes of xenografts of PC-9 (**d**) and PC-9/PEM clone1 (**f**). Data are means (SD), *n* = 6. **g** and **h** Changes in tumor volumes of xenografts at day 30 for PC-9 (**g**) and PC-9/PEM clone1 (**h**). Data are means (SD), *n* = 6. *, *P* < 0.05; **, *P* < 0.01, NS, not significant. Groups were compared by one-way ANOVA and Tukey’s HSD multiple comparisons test

To determine whether the combination treatment of defactinib and osimertinib affects normal cells in vitro, we examined viability of the normal fibroblast-like lung cells, OUS-11, after treating them with defactinib and osimertinib. In comparison to osimertinib treatment alone, the combination treatment did not increase cytotoxicity in OUS-11 cells (Fig. 5a). Furthermore, the combination treatment did not induce apoptosis in OUS-11 cells (Fig. 5b).

Next, to evaluate the antitumor activity of a combination of EGFR-TKI and a PTK2 inhibitor in vivo, we injected parental PC-9 or PC-9/PEM clone1 cells into the left or right flanks of nude mice. The mice were administered defactinib, osimertinib, a drug combination, or vehicle control by oral gavage 5 days per week. Tumor volumes are shown in Fig. 5c and e and the relative changes in tumor volume over time are shown in Fig. 5d and f. Comparing both cell lines, PC-9/PEM clone1 cells less grown than parental PC-9 on in vivo condition. The tumor volumes decreased in the combination drug treatment groups but increased in the vehicle- and defactinib-only-treated groups. No significant difference in relative PC-9 tumor volume was identified between osimertinib alone and the combination at day 30 (Fig. 5g). The combination treatment significantly (*P* < 0.01) inhibited the tumor growth in PC-9/PEM clone1 and reduced the tumor growth compared to day 0 (Fig. 5h). Pictures of mice are shown in Additional file 1: Figure S3A. No apparent adverse events such as weight loss were observed during these treatments (Additional file 1: Figure S3B). These data were consistent with those obtained from the in vitro experiments and suggested that defactinib restored in vivo EGFR-TKI sensitivity in the PC-9/PEM clone1 tumor.

### PTK2 is a therapeutic target against cells with acquired resistance to EGFR-TKI

**Fig. 6:**
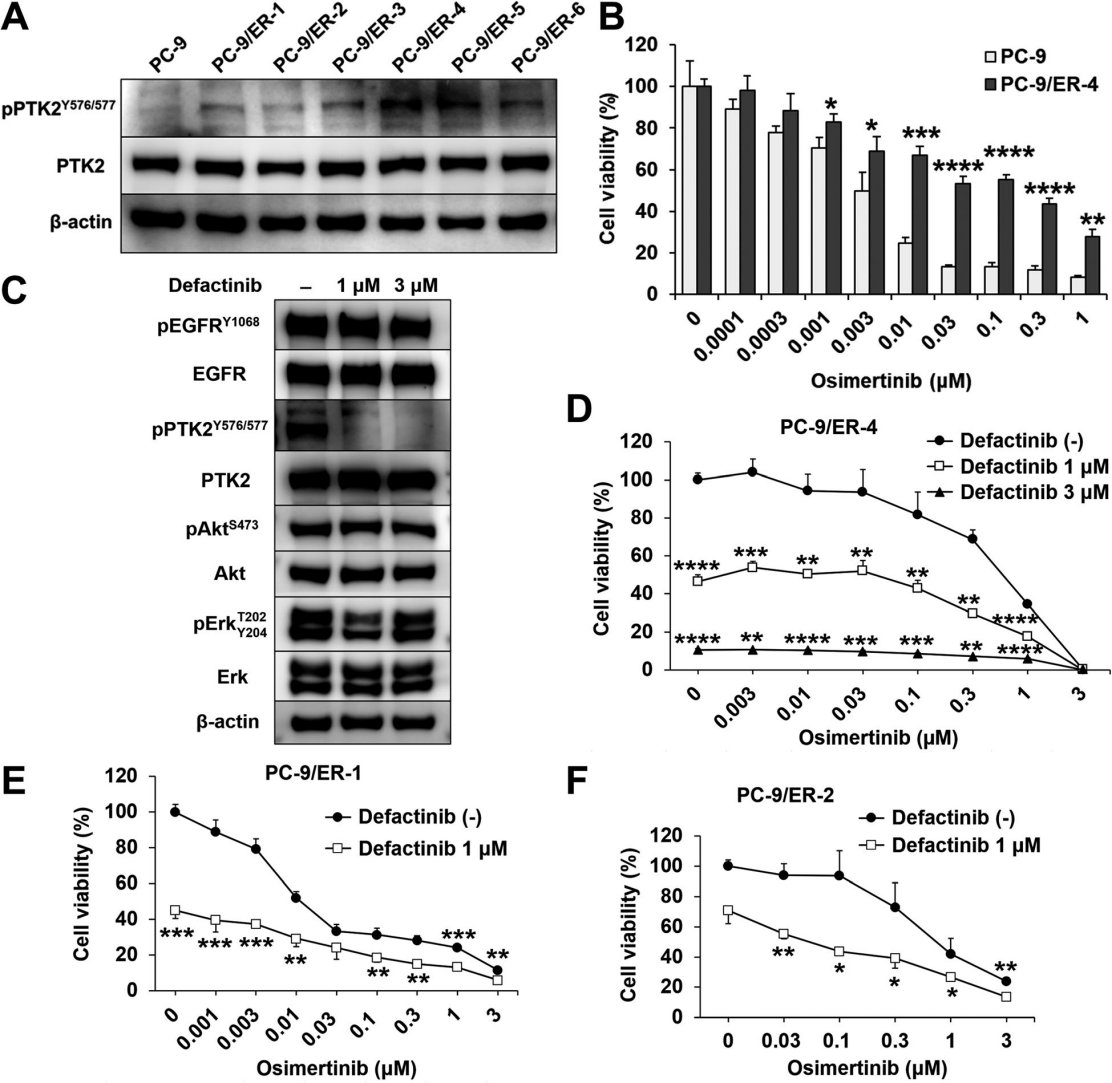
Defactinib and osimertinib co-treatment inhibited cell proliferation in various EGFR-TKI-resistant cell lines. **a** Immunoblots of phosphorylated and total PTK2 in PC-9 and PC-9/ER-1-6 cells. **b** Viabilities of PC-9 and PC-9/ER-4 cells treated with osimertinib at the indicated concentrations for 72 h. Data are means (SD), *n* = 3. *, *P* < 0.05; **, *P* < 0.01; ***, *P* < 0.001; ****, *P* < 0.0001. Data for treated and untreated cells were compared by Student’s t-test. **c** Immunoblots of the phosphorylated and total levels of the indicated proteins in PC-9/ER-4 cells treated with defactinib at the indicated concentration for 96 h. **d**-**f** Viability of PC-9/ER-4 cells with or without defactinib and osimertinib at the indicated concentrations for 96 h. Data are means (SD), *n* = 3. **, *P* < 0.01; ***, *P* < 0.001, ****, *P* < 0.0001. Data for untreated cells were compared with those for cells treated with defactinib by one-way ANOVA and Tukey’s HSD multiple comparisons test. Viabilities of PC-9/ER-1 (**e**) and PC-9/ER-2 (**f**) cells with or without 1 μM defactinib and osimertinib at the indicated concentrations for 96 h. Data are means (SD), *n* = 3. *, *P* < 0.05; **, *P* < 0.01; ***, *P* < 0.001 by Student’s t-test

EGFR-TKI-treated NSCLC may have acquired resistance to EGFR-TKIs via PTK2 hyperphosphorylation. We derived the erlotinib-resistant NSCLC cell lines PC-9/ER-1-6 from PC-9 (Additional file 1: Figure S4A-F). No T790 M mutation or *MET* amplification was detected in them (Additional file 1: Figure S4G-H). They expressed higher PTK2 phosphorylation than the parental PC-9 (Fig. 6a). PC-9/ER-4 had the highest PTK2 phosphorylation level of all erlotinib-resistant cell lines and was also resistant to osimertinib (Fig. 6b). Defactinib effectively inhibited PTK2 phosphorylation in PC-9/ER-4, but the phosphorylation of EGFR, Akt and Erk was not inhibited (Fig. 6c). A viability assay showed that defactinib was more effectively inhibit PC-9/ER-4 proliferation than osimertinib (Fig. 6d). Those results suggested that PC-9/ER-4 is addicted to PTK2 but not EGFR anymore to survive. In addition, defactinib with osimertinib effectively decreased the cell viability of PC-9/ER-1 than single osimertinib (Fig. 6e). There was an additive effect between defactinib and osimertinib against PC-9/ER-2 (Fig. 6f). Taken together, these results indicated that PTK2 hyperphosphorylation is correlated with acquired EGFR-TKI resistance. Furthermore, the combination of osimertinib and defactinib more effectively lowered EGFR-TKI-resistant NSCLC viability than osimertinib alone.

## Discussion

We have demonstrated that various EGFR-TKI-resistant NSCLC cell lines contained hyperphosphorylated PTK2. In addition, the combination of a PTK2 inhibitor and an EGFR-TKI resulted in a better therapeutic efficacy outcome for PTK2-activated EGFR-TKI-resistant cells than an EGFR-TKI alone in both in vitro and in vivo. Moreover, no adverse effects were observed for the combination in animal experiments. Therefore, PTK2 is a potential therapeutic target against EGFR-TKI-resistant NSCLC.

Relative to parental PC-9, the pemetrexed-resistant and erlotinib-resistant cell lines exhibited higher levels of PTK2 phosphorylation at the serial tyrosine sites (Y576/Y577) that activate the PTK2/Akt pathway [34, 35]. PTK2 upregulation and activation in tumors are linked to poor progression and aggressive disease [36, 37]. *PTK2* amplification in ovarian, head and neck, breast, and colorectal cancer may account for its overexpression in these tumors [28]. However, there was no significant difference in PTK2 expression between PC-9 and PC-9/PEM. Nevertheless, PTK2 protein levels may increase independently of *PTK2* gene expression [38]. *P53* mutation is highly correlated with PTK2 protein level in breast cancer cells [39]. No differences in PTK2 protein level were identified between parental PC-9 and EGFR-TKI-resistant cells in this study. Fang et al. reported that cells with a deletion variant at exon 33 of *PTK2* significantly phosphorylate PTK2 at the Y576/Y577 sites [40]. Though we did not find any variants at the *PTK2* exons in EGFR-TKI-resistant cell lines, certain transmembrane receptors such as integrins, growth factors, cytokine receptors, and G proteins could have activated PTK2 [41].

In the present study, the FGFR inhibitor PD173074 also repressed PTK2 (Fig. 4g). The PTK2 activation site was localized to the Y576/Y577 activation loop within the central domain of PTK2. Its phosphorylation is a PTK2 activation marker [41]. Phosphorylation of the p85 PI3K subunit on PTK2 triggers survival signaling by activating Akt. We found that PD173074 inhibits PTK2 phosphorylation at Y576/Y577. In fact, PTK2 and FGFR1 have the serial tyrosine phosphorylation sites Y576/Y577 and Y653/Y654, respectively, in the activation loop. Protein expression and phosphorylation are essential for kinase activity [30, 42, 43]; however, results from immunoblotting showed no FGFR1 protein expression in both PC-9 and PC-9/PEM (Additional file 1: Figure S1A-1C), and a decrease in PTK2 expression (Additional file 1: Figure S2M). Interestingly, sequence alignment by protein BLAST revealed the similarity of the structures of PTK2 and FGFR1 at the tyrosine sites (Additional file 1: Figure S5). Although activation of FGFR signaling may also contribute to acquired EGFR-TKI resistance in EGFR-mutant cancer [44]. The results suggest that PD173074 inhibits PTK2 directly but not FGFR1. Conversely, although we did not confirm the amount of the FGFR4 protein expression, no phosphorylation of FGFR4 in both PC-9 and PC-9/PEM was detected by immunoblotting (Additional file 1: Figure S1C). The FGFR4 inhibitor, BLU-554, did not inhibit the phosphorylation of PTK2 (Fig. 4g). Moreover, no additional growth inhibition was observed by the combination treatment of BLU-554 and an EGFR-TKI in comparison to the EGFR-TKI treatment alone (Additional file 1: Figure S2I-S2J). Even though FGFR4 mRNA expression was higher than that of FGFR1, these results indicate that FGFR4 protein was not activated in PC-9/PEM. While we did not measure the affinity of BLU-554 to PTK2, BLU-554 may not bind to PTK2 because BLU-554 is highly-specific to FGFR4 but not to FGFR1–3 [45, 46]. Therefore, we hypothesize that PTK2 directly confers EGFR-TKI resistance in PC-9/PEM without FGFR1 or FGFR4.

There has been a growing interest directed towards the use of PTK2 inhibitors in combination with existing therapeutic agents to enhance basic and clinical efficacy. A combination of VS-6063 (defactinib) and the PTK2 autophosphorylation inhibitor 1,2,4,5-benzenetetraamine tetrahydrochloride (Y15) synergistically decreased viability, clonogenicity, and attachment in thyroid cancer cell lines [26]. Several small-molecule PTK2 kinase inhibitors effectively inhibited tumor growth in various mouse xenograft models [47, 48]. Clinical studies showed that PTK2 is associated with tumor progression in various cancers. PTK2 upregulation is correlated with an aggressive phenotype of breast carcinoma [49]. In gastric cancer, PTK2 amplification is positively associated with age, tumor size, metastasis, and invasion [50]. A retrospective North American cohort study showed that PTK2 is expressed in greater than 50% of Stage I NSCLC cases but not in normal lung tissue [51]. Several orally bioavailable ATP-competitive PTK2 inhibitors have undergone clinical trials. Defactinib (VS-6063 or PF-04554878) is a second-generation PTK2 inhibitor. In a phase 1 study on Japanese patients with advanced solid tumors, defactinib was well tolerated at all dose levels by twice-daily administration and the area under the concentrations are within 5–10 μM on the time curve from time zero to 12 h [52]. This data suggested that a clinical dose of defactinib sufficiently inhibits hyperphosphorylated PTK2 comparing to our results of 3 μM defactinib in vitro. As the mechanisms of acquired resistance to osimertinib are gradually recognized with real-world data, novel therapeutic strategies for EGFR-mutated NSCLC have been explored in recent years. Immune-checkpoint therapy provides alternative for patients with solid tumors, combination use of EGFR-TKI and chemotherapy and use of fourth generation EGFR-TKI for the patients with EGFR C797S mutation [53]. Furthermore, combination treatments of EGFR-TKI and other targeted agents to inhibit bypass signaling such as AXL, MEK, PI3K, Akt, and mTOR are under evaluation in clinical trials [53, 54]. The combination of defactinib and other drugs such as pembrolizumab (ClinicalTrials.gov: NCT02758587), paclitaxel and carboplatin (ClinicalTrials.gov: NCT03287271), pembrolizumab and gemcitabine (ClinicalTrials.gov: NCT02546531) in patients with solid tumors were still under evaluation in clinical trials but no EGFR-TKI combination with defactinib at present [54]. Interestingly, PTK2 phosphorylation status at Y397 sites was reported to be associated with overall survival in NSCLC patients [55]. Thus, PTK2 and EGFR dual blockade should be considered for a clinical trial, especially involving EGFR-mutant NSCLC.

The mechanism of PTK2 activation remains unclear as no specific factor related to PTK2 activation was detected. We found no genetic variant that activated PTK2. Whole-genome sequencing may help to identify the variants responsible for heritable PTK2 activation in our EGFR-TKI-resistant cell lines. Therefore, we will analyze the genetic variants of PC-9/PEM clone1 by whole-genome sequencing. Larger animal studies are warranted to optimize treatment doses and assess combination treatment efficacy. We plan to evaluate clinical specimens for PTK2 phosphorylation as a target of PTK2 inhibitor treatment in NSCLC and translate this basic research into clinical trials. Specimens from patients treated with EGFR-TKI and whose clinical outcome has been assessed should be suitable for comparing clinical responses to EGFR-TKIs.

## Conclusions

Here, we provided evidence that PTK2 hyperphosphorylation is a critical factor in EGFR-TKI resistance in NSCLC. Moreover, we demonstrated that a combination of EGFR-TKI and PTK2 inhibitor is a potentially new therapeutic approach to overcome EGFR-TKI resistance.

## Supplementary information

**Additional file 1: Figure S1.** PC-9 and PC-9/PEM do not express FGFR1 or FGFR4 proteins. **Figure S2** PD173074 sensitized PC-9/PEM to erlotinib but BLU-554 and nintedanib did not. **Figure S3.** Representative pictures and body weights of xenografts. **Figure S4.** Establishment of erlotinib-resistant NSCLC cell lines. **Figure S5.** Sequence alignment between PTK2 (Y407 to F729) and FGFR1 (Y463 to L819). **Table S1.** The sequences for primers used in RT- qPCR.

## Data Availability

All data generated or analyzed during the current study are available from the corresponding author on reasonable request.

## Abbreviations

EGFR: Epidermal growth factor receptor
FGFR: Fibroblast growth factor receptor
NSCLC: Non-small cell lung cancer
PFS: Progression-free survival
PTK2: Protein tyrosine kinase 2
RT-qPCR: Reverse-transcription quantitative polymerase chain reaction
siRNA: Small interfering RNA
TKI: Tyrosine kinase inhibitor
