# Supplementary material for: Protein tyrosine kinase 2: a novel therapeutic target to overcome acquired EGFR-TKI resistance in non-small cell lung cancer
Source: Respir Res. 2019 Dec 2;20:270. doi: 10.1186/s12931-019-1244-2 (PMC6889213; doi:10.1186/s12931-019-1244-2)
Supplement: Supplementary file 1 — Additional file 1: Figure S1. PC-9 and PC-9/PEM do not express FGFR1 or FGFR4 proteins. Figure S2 PD173074 sensitized PC-9/PEM to erlotinib but BLU-554 and nintedanib did not. Figure S3. Representative pictures and body weights of xenografts. Figure S4. Establishment of erlotinib-resistant NSCLC cell lines. Figure S5. Sequence alignment between PTK2 (Y407 to F729) and FGFR1 (Y463 to L819). Table S1. The sequences for primers used in RT- qPCR. [file 12931_2019_1244_MOESM1_ESM.pdf]

Protein tyrosine kinase 2: A novel therapeutic target to overcome acquired EGFR-TKI resistance in non-small cell lung cancer

Xuexia Tong[1,2], Ryosuke Tanino[1], Rong Sun[1], Yukari Tsubata[1*], Tamio Okimoto[1], Mayumi Takechi[3], Takeshi Isobe[1]

[1]Department of Internal Medicine, Division of Medical Oncology & Respiratory Medicine, Faculty of Medicine, Shimane University, Shimane, Japan

[2]Department of Respiratory and Critical Care Medicine, General Hospital of Ningxia Medical University, Ningxia, China

[3]Department of Experimental Animals, Interdisciplinary Center for Science Research, Organization for Research and Academic Information, Shimane University, Shimane, Japan

Additional file 1

Figure S1 – PC-9 and PC-9/PEM do not express FGFR1 or FGFR4 proteins.

Figure S2 – PD173074 sensitized PC-9/PEM to erlotinib but BLU-554 and nintedanib did not.

Figure S3 – Representative pictures and body weights of xenografts.

Figure S4 – Establishment of erlotinib-resistant NSCLC cell lines.

Figure S5 – Sequence alignment between PTK2 (Y407 to F729) and FGFR1 (Y463 to L819).

Table S1 – The sequences for primers used in RT- qPCR.

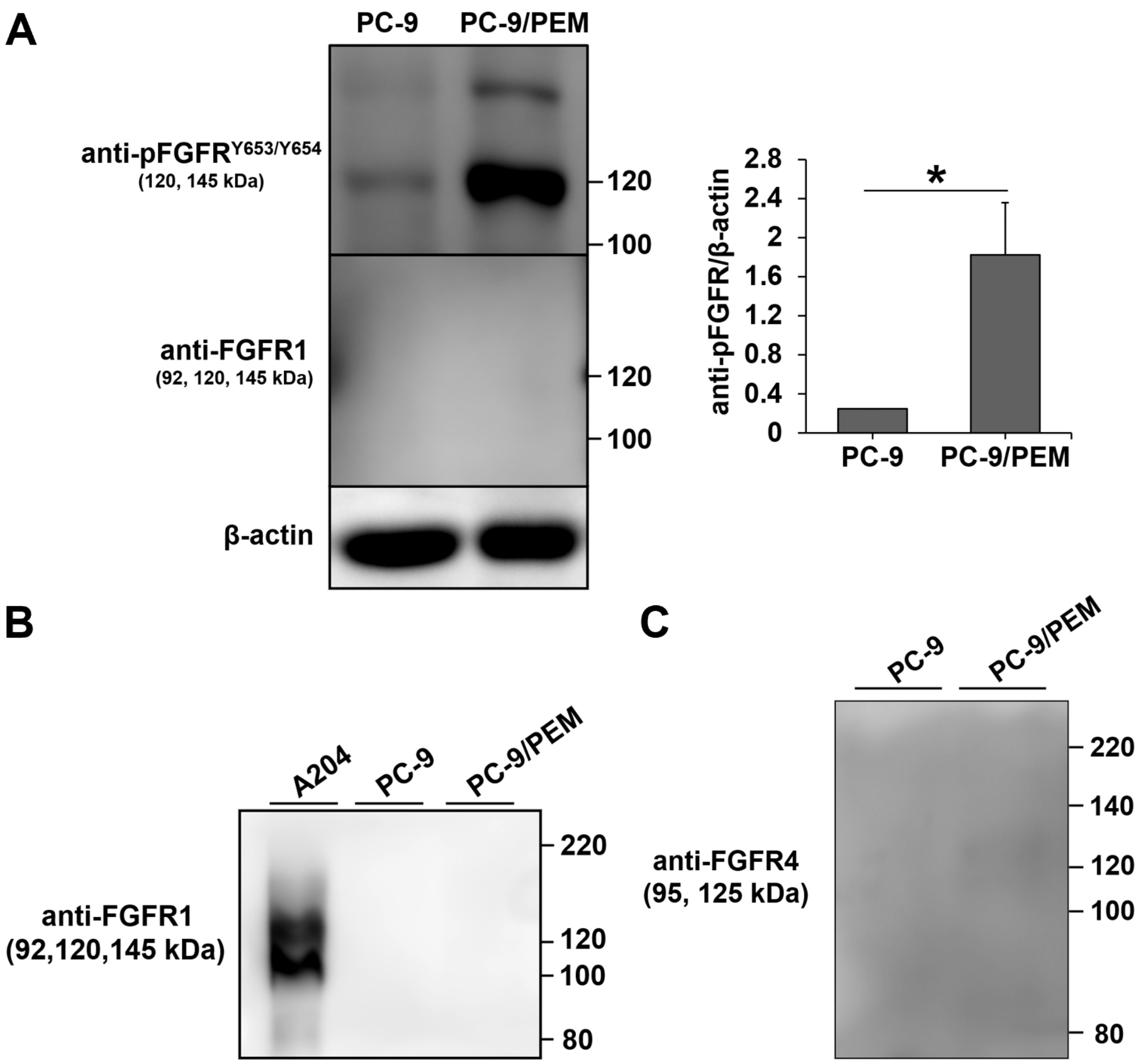

Figure S1 PC-9 and PC-9/PEM do not express FGFR1 or FGFR4 proteins. **A**, Representative immunoblots of protein binding to anti-pFGFR$^{Y653/Y654}$ and total FGFR1 in PC-9 and PC-9/PEM cells and ratio of phosphorylated protein to β-actin. Data are means (SD), n = 3. *, $P < 0.05$ by Student's *t*-test. **B**, Immunoblots of total FGFR1 in PC-9 and PC-9/PEM cells. A204 protein lysate was used as the positive control. **C**, immunoblots of total FGFR4 in PC-9 and PC-9/PEM cells.

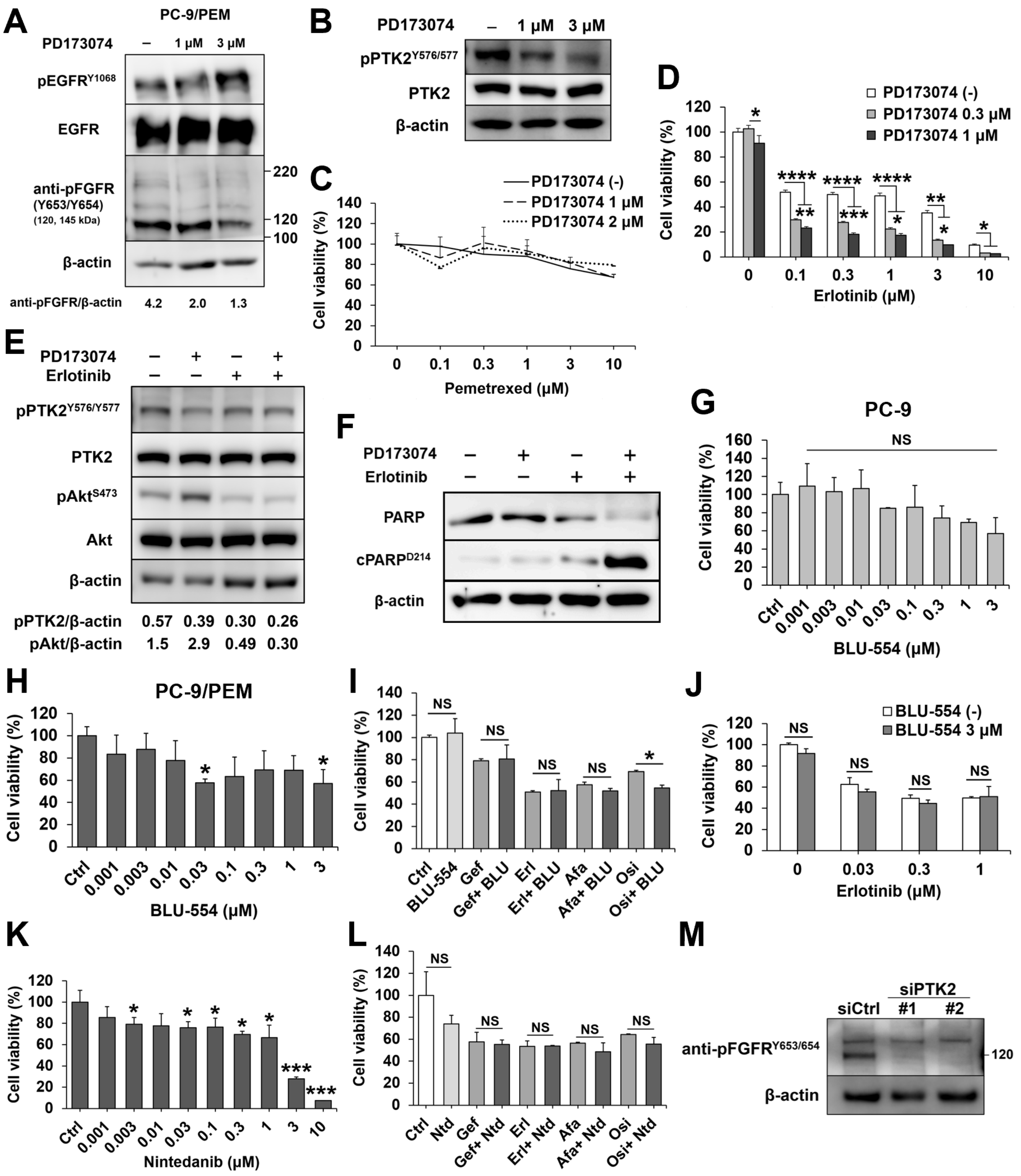

Figure S2 PD173074 sensitized PC-9/PEM to erlotinib but BLU-554 and nintedanib did not. **A**, Immunoblots of the phosphorylated and total EGFR, the protein binding to anti-pFGFR$^{Y653/Y654}$ and total FGFR1 in PC-9 and PC-9/PEM cells treated with PD173074 at the indicated concentration for 96 h. **B**, Immunoblots of phosphorylated- and total PTK2 in PC-9 and PC-9/PEM cells treated with the FGFR1 inhibitor PD173074 at the indicated concentration for 96 h. **C**, Viability of PC-

9/PEM cells with or without PD173074 and pemetrexed at the indicated concentrations for 72 h. Data are means (SD), n = 3. **D**, Viability of PC-9/PEM cells with or without PD173074 and erlotinib at the indicated concentrations for 96 h. Data are means (SD), n = 3. *, $P < 0.05$; **, $P < 0.01$; ****, $P < 0.0001$ by one-way ANOVA and Tukey's HSD multiple comparisons test. **E**, Immunoblots of the indicated proteins in PC-9 and PC-9/PEM cells with or without 1 μM PD173074 and 1 μM erlotinib for 72 h. **F**, Immunoblots of cleaved PARP and PARP in PC-9 and PC-9/PEM cells with or without 1 μM PD173074 and 1 μM erlotinib for 72 h. **G-H**, Viabilities of PC-9 (**G**) and PC-9/PEM (**H**) cells treated with the FGFR4 inhibitor BLU-554 at the indicated concentrations for 96 h. Data are means (SD), n = 3. *, $P < 0.05$; **, $P < 0.01$; ****, $P < 0.0001$. Data for treated and untreated cells were compared by one-way ANOVA and Tukey's HSD multiple comparisons test. **I**, viability of PC-9/PEM cells with or without 3 μM BLU-554 and 0.01 μM gefitinib (Gef), 1 μM erlotinib (Erl), 0.001 μM afatinib (Afa), or 0.01 μM osimertinib (Osi) for 96 h. Data are means (SD), n = 3. *, $P < 0.05$; NS, not significant by one-way ANOVA and Tukey's HSD multiple comparisons test. **J**, Viability of PC-9/PEM cells with or without BLU-554 and erlotinib at the indicated concentration for 96 h. Data are means (SD), n = 3. NS, not significant by Student's *t*-test. **K**, PC-9/PEM cells treated with the multi-kinase inhibitor nintedanib at the indicated concentrations for 96 h. Data are means (SD), n = 3. *, $P < 0.05$; ***, $P < 0.001$. Data for treated and untreated cells were compared by one-way ANOVA and Tukey's HSD multiple comparisons test. **L**, Viability of PC-9/PEM cells with or without 0.01 μM nintedanib (Ntd) and 0.01 μM gefitinib (Gef), 0.01 μM erlotinib (Erl), 0.0003 μM afatinib (Afa), or 0.03 μM osimertinib (Osi) for 96 h. Data are means (SD), n = 3. NS, not significant by one-way ANOVA and Tukey's HSD multiple comparisons test. **M**, Immunoblots of the protein binding to anti-pFGFR$^{Y653/Y654}$ in PC-9/PEM clone1 cells transfected with siRNAs against *PTK2*. The siCtrl was used as a negative control.

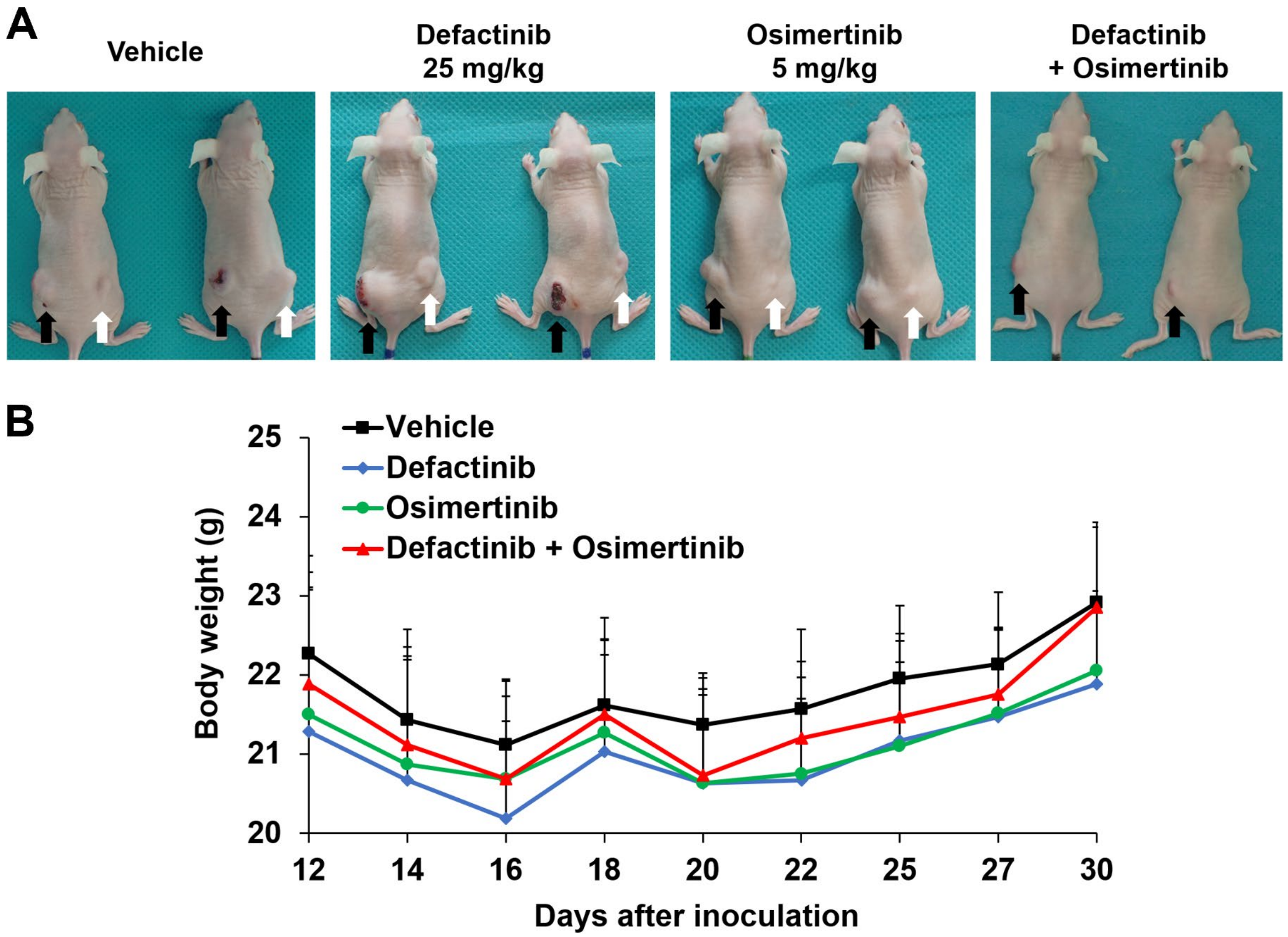

Figure S3 Representative pictures and body weights of xenografts. **A**, Xenografted mice with tumors at day 30. Black arrows indicate PC-9 tumors. White arrows indicate PC-9/PEM clone1 tumors. **B**, Body weights up to day 30. Data are means (SD); n = 6. Groups were compared by one-way ANOVA and Tukey's HSD multiple comparisons test.

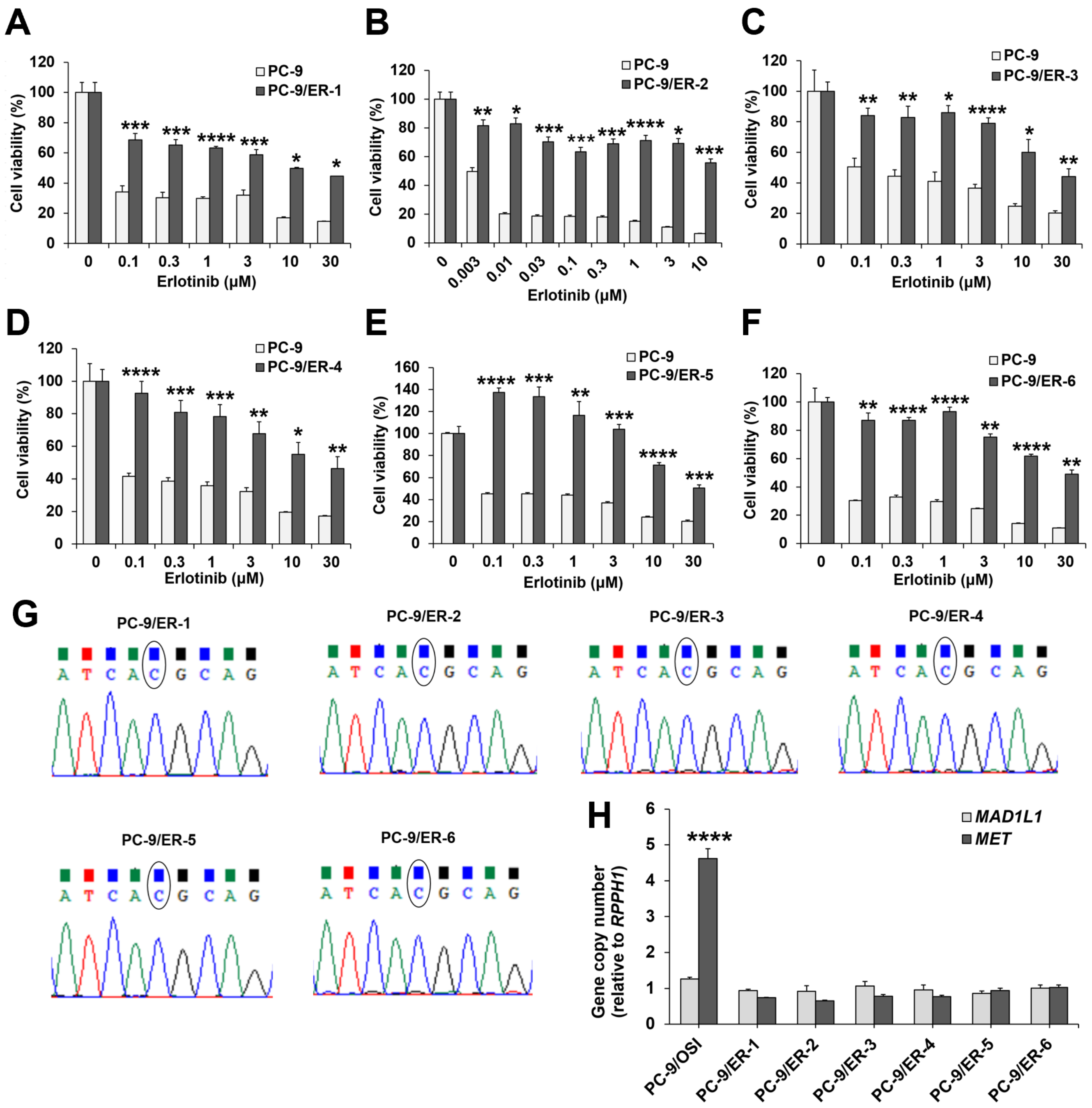

Figure S4 Establishment of erlotinib-resistant NSCLC cell lines. **A-F**, Viabilities of PC-9 and PC-9/ER-1 (**A**), PC-9/ER-2 (**B**), PC-9/ER-3 (**C**), PC-9/ER-4 (**D**), PC-9/ER-5 (**E**), and PC-9/ER-6 (**F**) cells treated with erlotinib at the indicated concentrations for 72 h. Data are means (SD), n = 3. *, $P < 0.05$; **, $P < 0.01$; ***, $P < 0.001$; ****, $P < 0.0001$. Data were compared with those for PC-9 cells by Student's *t*-test. **G**, Direct sequencing chromatogram at the p.T790 (c.2369C) site of EGFR in PC-9/ER-1 – 6. **H**, Copy number quantification of genomic DNA extracted from PC-9, PC-9/ER-1-6, and PC-9/OSI. *MET* or *MAD1L1* copy numbers relative to that for *RPPH1* are shown. *MAD1L1* was the negative control. Data are means (SD), n = 3. ****, $P < 0.0001$ by Student's *t*-test.

```
Range 1: 463 to 819  Graphics                                   ▼ Next Match  ▲ Previous Match

Score           Expect  Method                        Identities      Positives       Gaps
194 bits(494)   2e-55   Compositional matrix adjust.  120/357(34%)    187/357(52%)    34/357(9%)

Query  407  YTMPSTRDYEIQRERIELGRCIGEGQFGDV--HQGIYMSPENP--ALAVAIKTCKNCTSD  462
            Y +P    +E+ R+R+ LG+ +GEG FG V   + I +  + P     VA+K  K+  ++
Sbjct  463  YELPEDPRWELPRDRLVLGKPLGEGCFGQVVLAEAIGLDKDKPNRVTKVAVKMLKSDATE  522

Query  463  SVREKFLQEALTMRQF-DHPHIVKLIGVITEN-PVWIIMELCTLGELRSFLQVRK-----  515
                  + E   M+    H +I+ L+G  T++ P+++I+E  + G LR +LQ R+
Sbjct  523  KDLSDLISEMEMMKMIGKHKNIINLLGACTQDGPLYVIVEYASKGNLREYLQARRPPGLE  582

Query  516  ----------YSLDLASLILYAYQLSTALAYLESKRFVHRDIAARNVLVSSNDCVKLGDF  565
                        L    L+  AYQ++  + YL SK+ +HRD+AARNVLV+ ++ +K+ DF
Sbjct  583  YCYNPSHNPEEQLSSKDLVSCAYQVARGMEYLASKKCIHRDLAARNVLVTEDNVMKIADF  642

Query  566  GLSRYMEDSTYYK-ASKGKLPIKWMAPESINFRRFTSASDVWMFGVCMWEILMHGVKPFQ  624
            GL+R +    YYK  + G+LP+KWMAPE++  R +T  SDVW FGV +WEI   G  P+
Sbjct  643  GLARDIHHIDYYKKTTNGRLPVKWMAPEALFDRIYTHQSDVWSFGVLLWEIFTLGGSPYP  702

Query  625  GVKNNDVIGRIENGERLPMPPNCPPTLYSLMTKCWAYDPSRRPRFTELKAQLSTILEEEK  684
            GV   ++   ++ G R+  P NC   LY +M  CW   PS+RP F +L   L  I+
Sbjct  703  GVPVEELFKLLKEGHRMDKPSNCTNELYMMMRDCWHAVPSQRPTFKQLVEDLDRIVALTS  762

Query  685  AQQEERMRM---------ESRRQATVSW--DSGGSDEA-PPKPSRPGYPSPRSSEGF  729
             Q+   + M            R +T S   DS  S E  P +P  P +P+  ++ G
Sbjct  763  NQEYLDLSMPLDQYSPSFPDTRSSTCSSGEDSVFSHEPLPEEPCLPRHPAQLANGGL  819

Query:  PTK2 (Q05397)   Y576/Y577
Sbjct:  FGFR1 (P11362)  Y653/Y654
```

Figure S5 Sequence alignment between PTK2 (Y407 to F729) and FGFR1 (Y463 to L819). Blue indicates serial tyrosine sites of PTK2 and FGFR1.

Table S1 The sequences for primers used in RT- qPCR.

Gene	Forward primer (5′ to 3′)	Reverse primer (5′ to 3′)
IGF-1R	TGGTGGAGAACGACCATATCC	CGATTAACTGAGAAGAGGAGTTCGA
PXN	ACGTCTACAGCTTCCCCAACAA	AGCAGGCGGTCGAGTTCA
ITGβ1	CATCTGCGAGTGTGGTGTCT	AAGGCTCTGCACTGAACACA
Gab1	ATCAGAAACGCCAGCGAAGA	TCAGATACCACAAAGCACCA
Gab2	ACAGTACCTACGACCTCCCC	CTGGGCGTCTTGAAGGTGTA
Jak1	AGACTTGTGAATACGTTAAAAGAAGGA	AAAGCTTGTCCGATTGGATG
ErBb2	TGTGACTGCCTGTCCCTACAA	CCAGACCATAGCACACTCGG
PDGFRβ	GCACCGAAACAAACACACCTT	ATGTAACCACCGTCGCTCTC
MET	CCATCCAGTGTCTCCAGAAGTG	TTCCCAGTGATAACCAGTGTGTAG
MER	ACAGGTTCGGGACGTCCATC	CCGGGAATAGCGGGTAAGGC
PIK3CA	AATGCTTGGGGTGGAAGGGAC	GGGGTGCAAAACAATGCATGAC
AXL	TACCGCCAGGGACGTATCGC	CCAGCACCGCGACATCAAGG
GAS6	TGGCGCGGAATCTGGTCATC	GAAGCACTGCATCCTCGTGTTC
PDPK1	CAGAGGTCAGGCAGCAACATAGAG	ACGTCCTGTTAGGCGTGTGG
EPHA2	CCGGCTACACTGCCATCGAG	GCCCAGCATCCCTGGTCATC
TYRO3	AACATCTTGGGCCAGCTGTCTG	GATTTGGTCAGTCCGGGCTTC
PTEN	GACCCACACGACGGGAAGAC	GCCTCTGGATTTGACGGCTCC
TWIST1	CATGTCCGCGTCCCACTAG	TGTCCATTTTCTCCTTCTCTGG
PTK2	GCCTTAACAATGCGTCAGTTTGACC	AAATGACCTCAGCTCTCCAAGTGTG
FGFR1	GCATCATAATGGACTCTGTGGTG	GTGGTTGATGCTGCCGTACTC
FGFR2	CGCTGGTGAGGATAACAACACG	TTCCGCCATGACCACTTGCC
FGFR3	TACCGTGCTCAAGGTGTCCC	TTGCAGGTGTCGAAGGAGTAGTC
FGFR4	GGGGAGAACCGCATTGGAGG	ACACGTTCCGCAGGTACAGG
GAPDH	GCACCGTCAAGGCTGAGAAC	TGGTGAAGACGCCAGTGGA
